# Amino acid permease 3 (*aap3*) coding sequence as a target for *Leishmania* identification and diagnosis of leishmaniases using high resolution melting analysis

**DOI:** 10.1186/s13071-018-2989-z

**Published:** 2018-07-16

**Authors:** Karl Erik Müller, Ricardo Andrade Zampieri, Juliana Ide Aoki, Sandra Marcia Muxel, Audun Helge Nerland, Lucile Maria Floeter-Winter

**Affiliations:** 1 Department of Clinical Science, Faculty of Medicine, University of Bergen, Postboks 7804, 5020 Bergen, Norway; 2 Department of Physiology, Institute of Biosciences, University of São Paulo, Rua do Matão Travessa 14 no. 101, São Paulo, SP 05508900 Brazil

**Keywords:** PCR, HRM, Infectious diseases, *Leishmania* discrimination

## Abstract

**Background:**

The leishmaniases comprise a spectrum of clinical manifestations caused by different species of *Leishmania*. Identification of species is important for diagnosis, treatment and follow-up management. However, there is no gold standard for species identification. High resolution melting analysis (HRM) offers a possibility to differentiate *Leishmania* species without the need for processing of the PCR-product. The amino acid permease 3 (*aap3*) gene is an exclusive target for trypanosomatids and is conserved among *Leishmania* spp., thus it can be a valuable target for an HRM assay for diagnosis of the leishmaniases.

**Results:**

The HRM dissociation profiles of three amplicons targeting the *aap3-*coding region allowed the discrimination of *L.* (*Leishmania*) *donovani*, *L.* (*L*.) *infantum*, *L.* (*L*.) *major*, *L.* (*L*.) *tropica*, *L.* (*L*.) *mexicana, L.* (*L*.) *amazonensis*, *L.* (*Viannia*) *braziliensis*, *L.* (*V*.) *guyanensis*, *L.* (*V*.) *lainsoni*, *L.* (*V*.) *naiffi* and *L.* (*V*.) *shawi* using DNA from promastigote cultures*.* The protocol was validated with DNA samples from clinical infection in humans and a cat, naturally infected sand flies, and experimentally infected mice.

**Conclusions:**

HRM analysis using the *aap3* coding sequence as target is a relatively cheap, fast and robust strategy to detect and discriminate *Leishmania* species from all the endemic regions worldwide. The target and method proved to be useful in clinical, field and experimental samples, thus it could be used as a tool in diagnosis as well as ecological and epidemiological studies.

**Electronic supplementary material:**

The online version of this article (10.1186/s13071-018-2989-z) contains supplementary material, which is available to authorized users.

## Background

The leishmaniases are a group of diseases caused by *Leishmania* spp. Clinical presentations range from self-healing cutaneous lesions to potentially lethal visceral leishmaniasis [1]. It is defined by the World Health Organization (WHO) as a neglected tropical disease, meaning it is underreported, underestimated, underfunded and underprioritized by the pharmaceutical industry and often by public health authorities alike [2]. Over 350 million people are at risk of being infected, and it is estimated that 20,000 to 40,000 die each year of the leishmaniases [3]. In some regions, it is considered as largely an anthroponotic disease, while in others it is a zoonosis. The reservoir of the parasite can be domestic, sylvatic, and in some areas, both. Over 20 different species of *Leishmania* can cause the disease in humans [1, 4]. The species that cause disease in humans are grouped into two subgenera: *Leishmania* (*Leishmania*) and *Leishmania* (*Viannia*), based on biological features of the parasites [5]*.* Species identification is important for clinical diagnosis, prognosis, treatment and follow-up management. For example, some *L.* (*Viannia*) species, such as *L.* (*V*.) *braziliensis*, are known to cause cutaneous lesions and may later reappear as mucocutaneous lesions, thus requiring systemic treatment, while other lesions infected by strains known to only cause cutaneous manifestations may be treated with local treatment or observation therapy [6].

The leishmaniases can be diagnosed in various ways, all with their strengths and limitations. However, there is no gold standard for diagnosing the diseases. Microscopy is useful in a high-endemic setting, but lacks sensitivity and needs a trained microscopist, not easily found in most non-endemic areas. Diagnostic tests involving serology, such as the direct agglutination test (DAT) and lateral flow immunochromatographic tests can also be useful. Especially lateral flow immunochromatographic tests can give a rapid diagnosis, are simple to use, easy to interpret and relatively cheap - all qualities which are very important in a low-resource setting in many endemic areas. However, serological tests may vary greatly in their sensitivity and specificity between endemic regions [7]. Furthermore, serology is not able to determine the species causing the disease. Diagnosis involving nucleic acid detection is valuable due the high sensitivity and specificity, and for the potential ability to quantify and identify the infecting species. There is a plethora of possible techniques and an equal amount of possible targets [8].

Real-time PCR, followed by high-resolution melting analysis (HRM), generates thermodynamic differences in the dissociation profile of amplicons resulting in specific signatures of polymorphisms due to small differences in nucleotide composition [9]. HRM is rapid, comparatively little laborious and a relatively cheap method where the post-PCR treatment is contained in the tubes with small risk for lab-contamination. HRM has been used for identification of other infectious agents [10–13]. In earlier work, we have already shown that HRM can be a valuable tool for *Leishmania* genotyping, using *hsp70* as a target [14]. AAP3 is an amino acid transporter, which mediates uptake of lysine, histidine, phenylalanine, citrulline and arginine, with the highest affinity for the last one [15–20]. AAP3 is involved in the polyamine pathway, essential for parasite replication [17, 21–23]. The coding sequence for AAP3 is conserved among *Leishmania* spp., indicating its value as the chosen target [17]. We already demonstrated *aap3* as an attractive target for detecting *Leishmania*, by a real-time PCR method, but this approach did not discriminate the species [24]. In this paper, we describe the use of the *aap3* coding sequence as target for differentiation of *Leishmania* spp*.*, using HRM analysis. The *aap3*-HRM method showed to be a specific and sensitive tool to differentiate *Leishmania* spp., using reference strain cultures and validated using clinical samples, naturally infected sand flies and experimentally infected mice samples.

## Methods

### Organisms

Promastigotes of *Leishmania* spp. (see Table 1) were grown at 25 °C in M199 medium containing Earle’s salts, supplemented with 10% fetal bovine serum, 40 mM HEPES (pH 7.4), 100 μM adenine, 5 mg/l hemin, 0.05 mg/ml streptomycin and 4550 U/ml penicillin. *Trypanosoma cruzi* (Y-strain), *Crithidia fasciculata* (TCC-039) and *Endotrypanum schaudinni* (MCHO/BR/80/M6159) were grown at 28 °C in liver-tryptose medium supplemented with 10% fetal bovine serum and 0.05 mg/ml streptomycin and 4550 U/ml penicillin. *Trypanosoma brucei* (Lister 427) was grown at 28 °C in SDM-79 medium (LGC Biotecnologia, Cotia, São Paulo, Brazil), supplemented with 10% fetal bovine serum, 0.05 mg/ml streptomycin and 4550 U/ml penicillin. Mammalian DNA from BALB/c mouse and Wistar rat were obtained from the DNA repository of the Laboratory of Trypanosomatidae at Physiology - IB-USP and were used as negative controls (see Additional file 1: Figure S1 and Additional file 2: Figure S2).

**Table 1:** *Leishmania* reference strains and additional strains used in this study with information about international code number, clinical form and host according to the World Health Organization classification [25]

Strain	International code number	Clinical form	Isolated from	WHO reference strain
Assay standard strains				
*L.* (*L*.) *donovani*	MHOM/IN/80/DD8	Visceral	*Homo sapiens*	Yes
*L.* (*L*.) *infantum*	MCER/BR/1981/M6445	Visceral	*Cerdocyon thous*	No
*L.* (*L*.) *tropica*	MHOM/SU/60/OD	Cutaneous	*Homo sapiens*	No
*L.* (*L*.) *major*	MHOM/IL/81/Friedlin	Cutaneous	*Homo sapiens*	No
*L.* (*L*.) *amazonensis*	MHOM/BR/1973/M2269	Cutaneous	*Homo sapiens*	Yes
*L.* (*L*.) *mexicana*	MNYC/BZ/62/M379	Cutaneous	*Nyctomys sumichrasti*	Yes
*L.* (*V*.) *lainsoni*	MHOM/BR/81/M6426	Cutaneous	*Homo sapiens*	Yes
*L.* (*V*.) *braziliensis*	MHOM/BR/1975/M2903	Cutaneous	*Homo sapiens*	Yes
*L*. (*V*.) *guyanensis*	MHOM/BR/1975/M4147	Cutaneous	*Homo sapiens*	Yes
*L*. (*V*.) *naiffi*	MDAS/BR/1979/M5533	na	*Dasypus novemcinctus*	Yes
*L*. (*V*.) *shawi*	MCEB/BR/84/M8408	Cutaneous	*Cebus apella*	Yes
Additional strains used for specificity studies				
*L.* (*L*.) *donovani*	MHOM/CY/2006/CH33	Visceral	*Homo sapiens*	No
*L*. (*L*.) *tropica*	MHOM/SU/74/K27	Cutaneous	*Homo sapiens*	Yes
*L*. (*L*.) *tropica*	MHOM/MA/2000/INHW10	Cutaneous	*Homo sapiens*	No
*L*. (*L*.) *major*	MHOM/AF/2006/LEM5344	Cutaneous	*Homo sapiens*	No
*L*. (*L*.) *major*	MHOM/MA/2004/LEM4905	Cutaneous	*Homo sapiens*	No
*L*. (*L*.) *major*	MHOM/TN/2006/LPN296	Cutaneous	*Homo sapiens*	No
*L*. (*L*.) *major*	MRHO/SU/59/LV39	na	*Rhombomys opimus*	No
*L*. (*L*.) *mexicana*	MHOM/BZ/82/BEL21	Cutaneous	*Homo sapiens*	Yes
*L*. (*L*.) *mexicana*	MHOM/MX/93/CRE47	Cutaneous	*Homo sapiens*	No
*L*. (*L*.) *mexicana*	MHOM/MX/96/NAN01	Cutaneous	*Homo sapiens*	No
*L*. (*V*.) *braziliensis*	MHOM/BR/75/M2904	Cutaneous	*Homo sapiens*	Yes
*L*. (*V*.) *braziliensis*	MHOM/BR/87/LTB12MAR87	Mucocutaneous	*Homo sapiens*	No
*L*. (*V*.) *guyanensis*	MHOM/GF/94/22319	Cutaneous	*Homo sapiens*	No
*L*. (*V*.) *naiffi*	MHOM/GF/97/CRE88	Cutaneous	*Homo sapiens*	No

*Abbreviation*: *na* not applicable

### Naturally and experimentally infected samples

To validate the standardized protocols, samples previously identified by other diagnosis tests for the leishmaniases were used as a template in *aap3*-HRM assays [14, 26]. Human paraffin-embedded samples (from patients from Hospital das Clínicas da Universidade de São Paulo or Hospital da Irmandade da Santa Casa de Misericórdia de São Paulo), a sample from an infected cat (from Instituto de Medicina Tropical de São Paulo - USP), naturally infected sand flies (from Superintendência de Controle de Endemias de São Paulo), and samples from experimentally infected mice (from Instituto de Biociências - USP) were included.

### DNA extraction

The *Leishmania* strains used as assay-standards, *T. cruzi*, *T. brucei*, *C. fasciculata*, *E. schaudinni* and the mammalian DNA was isolated using a modified salting out technique, as previously described [14]. The DNA from the additional strains used for specificity studies was isolated using the QIAamp DNA Blood Mini Kit (Qiagen, Hilden, Germany), according to the manufacturer’s instructions. DNA from mouse was isolated from whole blood and DNA from rat was isolated from liver-tissue, both by using the DNeasy Blood and Tissue Kit (Qiagen), according to the manufacturer's instructions. DNA quality and concentration were determined by NanoDrop ND-1000 Spectrophotometer (Thermo Scientific, USA). All DNA was stored at -20 °C until further use.

For the paraffin-embedded tissues, a first step of washing was performed with xylol heated to 95 °C to remove paraffin, followed by repetitive washes of absolute ethanol. Then DNA was purified by an organic extraction with phenol-chloroform according Uliana et al. [27]. For sand flies, cultured *Leishmania* and mice samples, DNA was purified by silica columns, using DNeasy Blood & Tissue Kit (Qiagen).

### Primer design

The primers (listed in the Table 2) were designed based on the following sequences: *L.* (*L*.) *amazonensis* (HQ912026.1 and HQ912027.1), *L.* (*V*.) *braziliensis* (XM_001567050.2), *L.* (*L*.) *major* (XM_001685021.1), *L.* (*L*.) *donovani* (AY247004.1), *L.* (*L*.) *infantum* (XM_001467313.2) from GenBank, and *L.* (*L*.) *aethiopica* (LAEL147_000015800), *L.* (*L*.) *mexicana* (L.mxM.30.0870), *L.* (*L*.) *tropica* (LTRL590_310015200) and *L.* (*V*.) *panamensis* (LPAL13_000030300) from the TriTryp Database [28]. To predict melting temperatures (Tm), Oligo Calculator version 3.27 was used [29]. The nucleotide sequences of the amplicons and primer localizations are detailed in Additional file 3. A global BLAST search was performed for the three amplicon regions using the available amplicon sequences from the strains listed above.

**Table 2:** Primer sequences used in this study based on the amino acid permease 3 (*aap3*) coding sequence, for amplicon 1 (Amp1), amplicon 2 (Amp2) and amplicon 3 (Amp3)

Name	Sequence (5'-3')	Length (bp)	Orientation	Amplicon size (bp)
AAP3-Amp1-F	ATCCGCTACGTCTCCGCCATCGG	23	Forward	123
AAP3-Amp1-R	CGTGGTGAAGTACTTCATGTCGC	23	Reverse	
AAP3-Amp2-F	GCCGTCGATAAACACCCGAGC	21	Forward	131
AAP3-Amp2-R	AAGCGGAAGATGATGTTGCGCCC	23	Reverse	
AAP3-Amp3-F	GGCGGTCGCCTACATCAGCG	20	Forward	140
AAP3-Amp3-R	CGGGCACCATGAACACGAGCCATA	24	Reverse	

### PCR assays

Conventional PCR with the primers (Table 2) was performed for testing amplicon specificity. PCR reactions were performed using TopTaq Master Mix (Qiagen, Hilden, Germany) in a final volume of 25 μl with 200 nM of each primer and 25 ng of genomic DNA as a template. The cycling conditions were as follows: initial denaturation at 94 °C for 3 min, followed by 25 cycles of denaturation at 94 °C for 1 min, annealing at 60 °C for 1 min and extension at 72 °C for 30 s and a final extension at 72 °C for 10 min.

Real-time PCR was performed using MeltDoctor HRM Master Mix (Applied Biosystems, Foster City, CA, USA), according to the manufacturer’s instructions. Final reaction volume was 20 μl, including 200 nM of each primer and 25 ng of genomic DNA as a template. Real time amplification conditions were as follows: an initial denaturation step at 95 °C for 5 min, followed by 40 cycles of denaturation at 94 °C for 30 s and annealing/extension at 60 °C for 1 min, with the acquisition of fluorescent signals at the end of each extension step, followed by the dissociation curve for HRM analysis. All reactions were performed in a Thermocycler PikoReal96 (Thermo Fisher Scientific, Walthan, MA, USA).

### High resolution melting analysis

For HRM analysis, fluorescent signals were detected at 0.2 °C intervals, with hold-time for 10 s, between 60–95 °C. Data analysis was performed using PikoReal 2.1 Software (Thermo Fisher Scientific, Walthan, MA, USA).

### Sensitivity assays

For sensitivity assays we tested the performance of the three amplicons with standard strains which produced an overlapped melting profile (see Table 1 for information about the strains).

For amplicon 1, we tested *L.* (*L*.) *donovani*, *L.* (*L*.) *mexicana, L.* (*V*.) *braziliensis* and *L.* (*V*.) *guyanensis.* For amplicon 2, we tested *L.* (*L*.) *donovani*, *L.* (*L*.) *mexicana*, *L.* (*L*.) *infantum* and *L.* (*L*.) *tropica*. For amplicon 3, we tested *L.* (*L*.) *donovani*, *L.* (*L*.) *mexicana*, *L.* (*V*.) *braziliensis* and *L.* (*V*.) *guyanensis*. For all amplicons, a range of parasite DNA from 25 ng to 100 fg was tested, with or without 25 ng/μl of human DNA. The DNA from standard strains was purified from *in vitro* cultivated parasites, as described above. Efficiency calculations were made for each amplicon from template ranging from 25 ng to 50 pg (see Additional file 4: Figure S3).

### Statistics

All samples were tested in duplicate in at least three independent experiments. One-way ANOVA was used to calculate statistical difference between the Tm’s of paired species for each amplicon. The results are presented as mean differences with 95% confidence intervals, and statistical significance set to *P* < 0.05. The results were analyzed and graphs were produced using GraphPad Prism version 7 (Additional file 5).

## Results

### Specificity

**Fig. 1:**
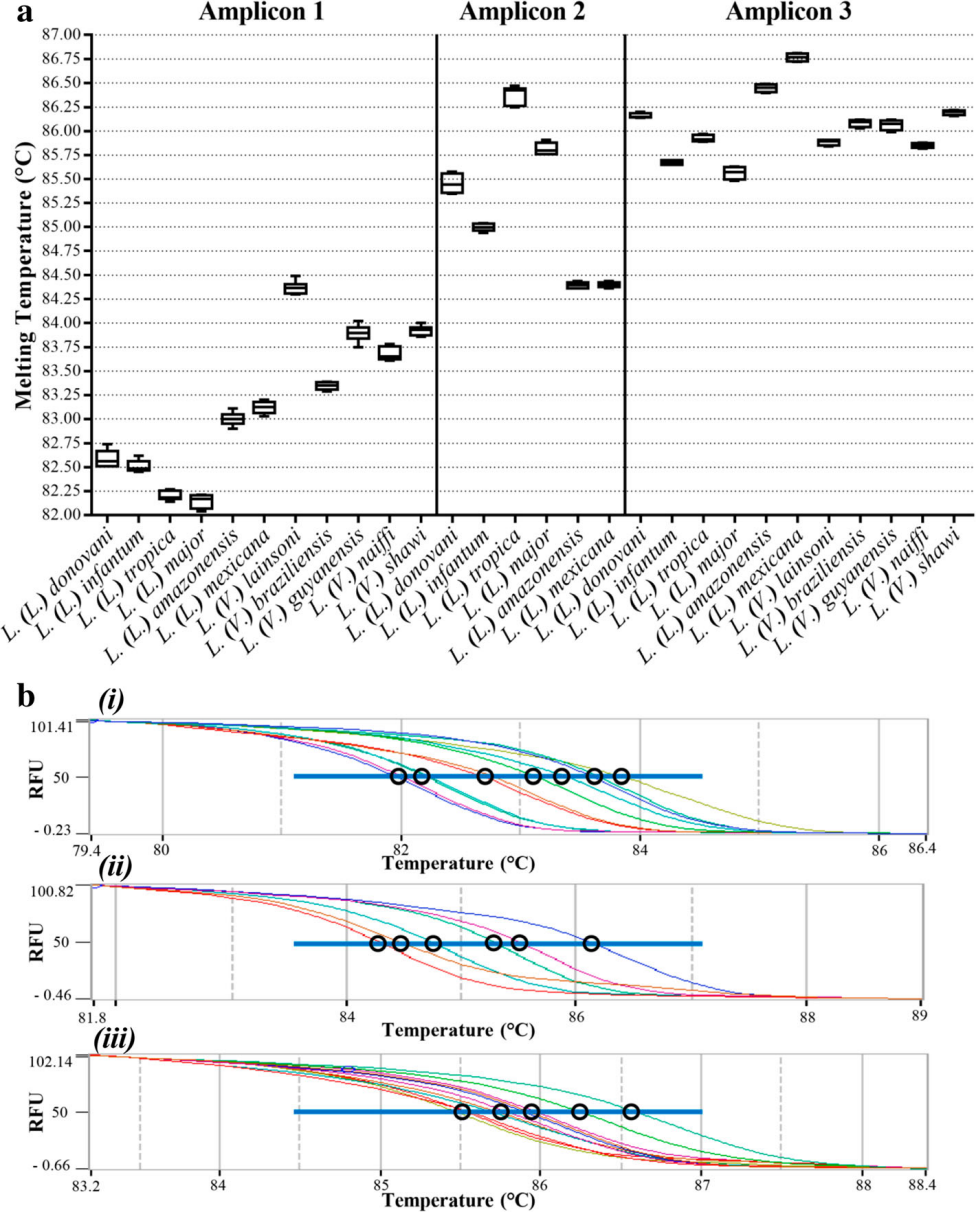
Melting temperatures and normalized melting profiles obtained with the HRM assays. **a** Melting temperatures (Tm) for all amplicons and standard species. For all amplicons 25 ng of genomic DNA was used as template. The plots show the mean, minimum and maximum of the Tm-values. Each species was tested in duplicate in three independent experiments. **b** Normalized HRM plots for all amplicons: *(i)*, *(ii)* and *(iii)* represent the normalized melting profile for amplicon 1, amplicon 2 and amplicon 3, respectively. Each sample was tested in duplicate in three independent experiments. For *(i)*, the bundles circled in the blue line, respectively from the left to the right, represent the profiles for *L.* (*L*.) *tropica*/*L.* (*L*.) *major*, *L.* (*L*.) *donovani*/*L.* (*L*.) *infantum*, *L.* (*L*.) *amazonensis*/*L.* (*L*.) *mexicana*, *L.* (*V*.) *braziliensis*, *L.* (*V*.) *naiffi*, *L.* (*V*.) *guyanensis*/*L.* (*V*.) *shawi* and *L.* (*V*.) *lainsoni*. For *(ii)*, the bundles circled in the blue line, respectively from the left to the right, represent the profiles for *L.* (*L*.) *amazonensis*, *L.* (*L*.) *mexicana*, *L.* (*L*.) *infantum*, *L.* (*L*.) *donovani*, *L.* (*L*.) *major* and *L.* (*L*.) *tropica*. For *(iii)*, bundles circled in the blue line, respectively from the left to the right, represent the profiles for *L.* (*L*.) *infantum*/*L.* (*L*.) *major*, *L.* (*L*.) *tropica*/*L.* (*V*.) *lainsoni*/*L.* (*V*.) *naiffi*, *L.* (*L*.) *donovani*/*L.* (*V*.) *braziliensis*/*L.* (*V*.) *guyanensis*/*L.* (*V*.) *shawi*, *L.* (*L*.) *amazonensis*, *L.* (*L*.) *mexicana*. *Abbreviation*: RFU: relative fluorescence units

Three amplicons were designed based on available sequences from GenBank or TriTryp database [28], considering that *aap3* is present in two copies situated *in tandem* around 4 kb apart in most of *Leishmania* species [16, 47]. As expected, the BLAST search did not reveal any other hits than for *Leishmania* spp. As shown in Additional file 1: Figure S1, designed primers generated amplicons of the same size for all the species, presenting differences in nucleotide composition. To predict and to delimit potentially informative polymorphic regions, theoretical melting temperatures were calculated *in silico* using the OligoCalc tool [29] (data not shown), to guide primer design. In addition, the two copies of the gene were aligned concomitantly for the choice of primers that amplify both copies (data not shown). Although some single nucleotide polymorphism (SNP’s) were detected between the two copies of the gene, it did not alter the Tm values. The alignments also allowed the selection of conserved regions for the design of oligonucleotides common to all species, which were used to generate products containing differences in nucleotide composition that were able to distinguish species or groups of species. Furthermore, we analyzed the amplification profile by conventional PCR using the same primers (Additional file 1: Figure S1). We observed specific products of the same size for all species analyzed for each set of primers: 123, 131 and 140 bp for amplicon 1, amplicon 2 and amplicon 3, respectively. No formation of dimers of primers were detected. Control samples were used and no amplification was observed for *T. brucei*, *T. cruzi*, *E. schaudinni*, rat, mouse and human DNA (Additional file 1 and Additional file 2). The analysis of amplicon 3 showed an amplification of *C. fasciculata*, a non-pathogenic and closely related organism to *Leishmania* spp., but the Cq and Tm values were able to distinguish *C. fasciculata* from all *Leishmania* spp. tested (Additional file 1: Figure S1 and Additional file 2: Figure S2 ). Individual Tm values and normalized melting profiles of 3 amplicons for reference strains are presented in Fig. 1, indicating that these differential profiles distinguished among *Leishmania* spp. To reliably distinguish different species, we only considered differences in Tm values exceeding ± 0.25 °C, an interval statistically determined as discriminatory (Additional file 5: Figure S3).

According to Tm and melting curves, amplicon 1 was able to distinguish the species into 3 clusters for subgenus *L.* (*Leishmania*): the visceral *L.* (*L*.) *donovani* and *L.* (*L*.) *infantum*, cutaneous strains from Eurasia and Africa [*L.* (*L*.) *major* and *L.* (*L*.) *tropica*], and cutaneous and mucocutaneous strains from the Americas *L.* (*L*.) *amazonensis, L.* (*L*.) *mexicana*, and strains of subgenus *L.* (*Viannia*). Furthermore, it was also able to distinguish some species of the subgenus *L.* (*Viannia*) into 3 clusters: *L.* (*V*.) *lainsoni*, *L.* (*V*.) *braziliensis* from the other species in that subgenus (Fig. 1).

Amplicon 2 was especially designed for strains of the subgenus *L*. (*Leishmania*). No amplification for subgenus *L.* (*Viannia*) was observed (Additional file 1: Figure S1). It was able to further distinguish the visceral strains into two different clusters: *L.* (*L.*) *donovani* and *L.* (*L*.) *infantum*. For the cutaneous species, it was able to distinguish the Eurasian and African cutaneous strains *L.* (*L*.) *major* and *L.* (*L*.) *tropica*. However, no difference in melting temperature was detected for the American cutaneous strains *L.* (*L*.) *amazonensis* and *L.* (*L*.) *mexicana* (Fig. 1). Therefore, amplicon 3 was designed for this purpose.

Amplicon 3 was, as predicted, able to further distinguish *L.* (*L*.) *amazonensis* from *L.* (*L*.) *mexicana*. Furthermore, it was able to distinguish *L.* (*L*.) *donovani* from *L.* (*L*.) *infantum*, thereby distinguishing the two species commonly causing VL. It was also able to distinguish the more common causes of CL in Eurasia and Africa: *L.* (*L*.) *major* and *L.* (*L*.) *tropica.*

Differences in amplification efficiencies could be observed when the amplification curves from real-time PCR were analyzed. Cq (quantification cycle) data were used as a relative parameter of quantification for the 3 targets when normalized amounts of samples were compared (see Additional file 2: Figure S2). Using 25 ng as template, for all *Leishmania* species, the amplification reactions produced curves with similar Cq values for amplicon 1 and 3, indicating a good specificity for all species. For amplicon 2, no amplification of DNA from subgenus *L.* (*Viannia*) was observed. In addition, high Cq-values for *L.* (*L*.) *amazonensis, L.* (*L*.) *major* and *L.* (*L*.) *mexicana* indicate a lower efficiency compared with the other *L.* (*Leishmania*) species, which also could be used as a parameter of discrimination. For amplicon 1, there was an unspecific amplification of human DNA. However, both the Cq and Tm values were distinct.

For all amplicons, additional strains were tested for specificity for all species, except *L.* (*L*.) *infantum*, *L.* (*L*.) *amazonensis*, *L.* (*V*.) *lainsoni* and *L.* (*V*.) *shawi*, as we did not have additional strains for these species available. For amplicon 1, we found that most strains, except *L.* (*L*.) *major* and *L.* (*V*.) *naiffi* showed a consistent profile for their melting temperatures. For amplicon 2 a relatively large SD was found for *L.* (*L*.) *donovani* (Table 3).

**Table 3:** Average melting temperatures and standard deviations (SD) for standard strains and additional strains (see Table 1 for further information on all strains tested)

Species	Amplicon 1		Amplicon 2		Amplicon 3	
	Average	SD	Average	SD	Average	SD
*L.* (*L*.) *donovani*	82.47	0.16	85.23	0.30	86.04	0.08
*L.* (*L*.) *infantum*	82.51	na	85.02	na	85.67	na
*L.* (*L*.) *tropica*	82.22	0.02	86.47	0.08	85.91	0.07
*L.* (*L*.) *major*	82.29	0.24	85.90	0.11	85.57	0.04
*L.* (*L*.) *amazonensis*	83.00	na	84.43	na	86.45	na
*L.* (*L*.) *mexicana*	83.12	0.00	84.46	0.05	86.88	0.09
*L*. (*V*.) *lainsoni*	84.39	na	na	na	85.88	na
*L*. (*V*.) *braziliensis*	83.44	0.18	na	na	85.97	0.04
*L*. (*V*.) *guyanensis*	83.83	0.15	na	na	86.14	0.11
*L*. (*V*.) *naiffi*	83.89	0.26	na	na	86.00	0.21
*L*. (*V*.) *shawi*	83.92	na	na	na	86.20	na

*Abbreviation*: *na* not applicable, where only one strain was tested

### Sensitivity

To evaluate the role of DNA-concentration, we tested the performance of the three amplicons in strains that presented close melting temperatures, based on the specificity assays. The limit of detection (LOD) was estimated using ten-times serial dilutions from 25 ng to 100 fg of DNA from standard strains purified from *in vitro* cultured parasites. For amplicon 1 the LOD was 100 fg for all species tested except *L.* (*L*.) *mexicana*, which had a LOD of 250 fg. For amplicon 2 the LOD was 100 fg for all species tested except for *L.* (*L*.) *mexicana*, which had a LOD of 50 pg. For amplicon 3, the LOD was lower for all species, with 500 fg for *L.* (*L*.) *donovani*, *L.* (*L*.) *mexicana* and *L.* (*L*.) *guyanensis*, while for *L.* (*V*.) *braziliensis* the LOD was 50 pg. The LOD was the same for all amplicons and all species in mixtures of *Leishmania* and human DNA (data not shown). Considering that the *Leishmania* genome sizes range between 29 Mb and 33 Mb, varying from 34 to 36 chromosomes [30], the estimated single-cell DNA is approximately 75 fg. Thus, we can assume that 100 fg is equivalent to just above one parasite in most of the *Leishmania* species.

**Fig. 2:**
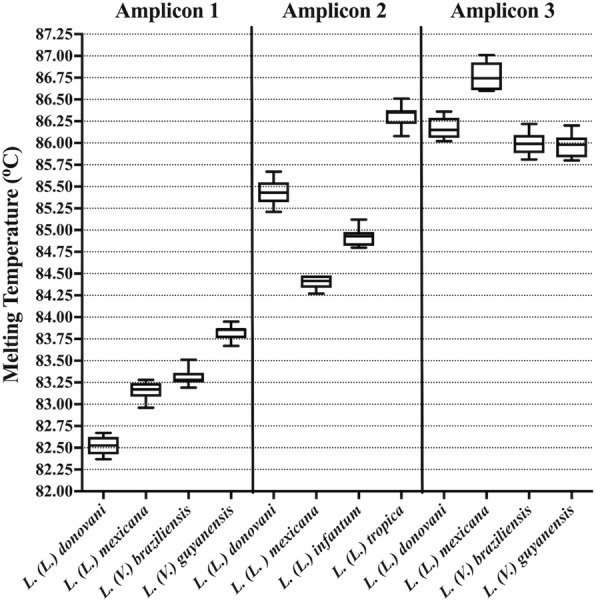
Effect of DNA-concentration on melting temperature (Tm °C). Dispersion graph of Tm values for amplicons 1, 2 and 3 from a range of 25 ng to 100 fg of DNA template. The plot shows mean, minimum and maximum Tm-values

We further tested if the initial amount of DNA would cause a variation in the melting temperature by serially diluting DNA from 25 ng to 100 fg with or without 25 ng/μl of human DNA. For all three amplicons, the results were the same with or without human DNA. There was a slight variation in melting temperature for all the species, only exceeding 0.2 °C for *L.* (*V*.) *guyanensis* in amplicon 1 with human DNA (Table 4). Furthermore, for amplicon 1 the difference in melting temperature between *L.* (*L*.) *mexicana* and *L.* (*V*.) *braziliensis* could potentially cause a confusion between these two species if melting temperatures were considered for this amplicon, the same can be noticed for *L.* (*L.*) *donovani, L.* (*L.*) *braziliensis* and *L.* (*L.*) *guyanensis* for amplicon 3 (Fig. 2).

**Table 4:** Effect of initial amount of DNA on melting temperature. Mean and standard deviation (SD) for serial dilutions of DNA from standard strains. DNA concentration ranged from 25 ng to 100 fg. Human DNA concentration was kept constant at 25 ng/μl

Strain	Without human DNA	With human DNA
	Mean Tm ± SD (°C)	Mean Tm ± SD (°C)
Amplicon 1		
*L.* (*L*.) *donovani*	82.52 ± 0.10	82.64 ± 0.18
*L.* (*L.*) *mexicana*	83.16 ± 0.10	83.23 ± 0.13
*L.* (*V*.) *braziliensis*	83.31 ± 0.09	83.43 ± 0.11
*L.* (*V*.) *guyanensis*	83.83 ± 0.08	83.96 ± 0.34
Amplicon 2		
*L.* (*L*.) *donovani*	85.44 ± 0.14	85.44 ± 0.14
*L.* (*L*.) *mexicana*	84.40 ± 0.08	84.56 ± 0.07
*L.* (*L*.) *infantum*	84.92 ± 0.11	85.17 ± 0.14
*L.* (*L*.) *tropica*	86.33 ± 0.13	86.73 ± 0.18
Amplicon 3		
*L.* (*L*.) *donovani*	86.17 ± 0.12	86.15 ± 0.12
*L.* (*L*.) *mexicana*	86.77 ± 0.16	86.91 ± 0.16
*L.* (*V*.) *braziliensis*	86.00 ± 0.14	86.17 ± 0.15
*L.* (*V*.) *guyanensis*	85.97 ± 0.13	86.36 ± 0.13

### Efficiency

The reaction efficiency for each amplicon was performed using 25 ng, 5 ng and 50 pg of DNA from each reference *Leishmania* species. Efficiency curves, slopes and *R*^2^ values were calculated (see Additional file 4: Figure S3). The template points produced Cq values between 20 and 30, where the PCR reactions produced copies of template at exponential ratios and with 95–105% of efficiency. The efficiency patterns were similar for all species testes for each amplicon, except for *L.* (*L*.) *mexicana* in amplicon 2. The lower efficiency for *L.* (*L*.) *mexicana* for amplicon 2 can be explained by the presence of mismatches comparing in primers (see Additional file 3), as can also be observed in the Cq values showed in Additional file 2: Figure S2.

### Validation using naturally and experimentally infected samples

To validate the *aap3*-HRM protocol with other samples than reference strain cultures, we applied this protocol in seven biological samples from naturally infected humans, naturally infected cat, naturally infected sand flies, and experimentally infected BALB/c mice (Table 5). The results of amplicon 1 showed a correlation with those obtained with other diagnostic targets; small subunit ribosomal DNA (SSU rDNA) [26], glucose-6-phosphate dehydrogenase (*g6pd*) PCR [31] and heat-shock protein 70 (*hsp70*) HRM [14]. Although amplicons 2 and 3 were not able to amplify all samples, the positive samples correlated with results obtained with other targets.

**Table 5:** Identification of *Leishmania* in naturally and experimentally infected samples by HRM analysis targeting the *aap3* gene

Sample source	HRM identification			Previous diagnosis	
	Amplicon 1	Amplicon 2	Amplicon 3	Diagnostic method	Species identification
Human^a^	*L.* (*L*.) *amazonensis*	negative	negative	SSU rDNA sequencing	*L.* (*L*.) *amazonensis*
Human^b^	*L.* (*L*.) *infantum*	*L.* (*L*.) *infantum*	*L.* (*L*.) *infantum*	SSU rDNA sequencing	*L.* (*L*.) *infantum*
Cat^c^	*L.* (*L*.) *infantum*	*L.* (*L*.) *infantum*	*L.* (*L*.) *infantum*	*hsp70* HRM	*L.* (*L*.) *infantum*
Mouse^d^	*L.* (*L*.) *amazonensis*	*L.* (*L*.) *amazonensis*	*L.* (*L*.) *amazonensis*	SSU rDNA sequencing	*L.* (*L*) *amazonensis*
Mouse^d^	*L.* (*V*.) *braziliensis*	negative	*L.* (*V*.) *braziliensis*	*g6pd* PCR	*L.* (*V*.) *braziliensis*
Sand flies^e^	*L.* (*L*.) *infantum*	*L.* (*L*.) *infantum*	*L.* (*L*.) *infantum*	SSU rDNA sequencing	*L.* (*L*.) *infantum*
Sand flies^f^	Subgenus *Viannia*	negative	Subgenus *Viannia*	*g6pd* PCR	*L.* (*V*.) *braziliensis*

*Note*: The *aap3* amplicons 1, 2 and 3 of DNA from each sample was submitted to HRM analysis. The result was compared with previous identification performed by SSU rDNA-sequencing [26], hsp70 HRM [14] or *g6pd* PCR [31]

^a^Human paraffin-embedded tissue from Hospital das Clínicas de São Paulo

^b^Human paraffin-embedded tissue from Irmandade da Santa Casa de Misericórdia de São Paulo

^c^Isolated parasites from cat

^d^Experimentally infected BALB/c mice

^e^Naturally infected *Lutzomyia* (*Lutzomyia*) *longipalpis*

^f^Naturally infected *Lu.* (*Nyssomyia*) *whitmani*

## Discussion

Diagnosing *Leishmania* infection at the species level is important, as it may guide treatment options and follow-up protocols. Accurate diagnosis is also important in an ecological and epidemiological sense. Unfortunately, in most endemic regions, the leishmaniases are underreported, and the true burden of the disease remains unknown. Techniques that target genomic or mitochondrial DNA by PCR or related techniques are today the most common for detection and identification of *Leishmania* spp*.* However, there is no gold standard in methods and targets [8]. Common targets such as kinetoplast DNA (kDNA) and SSU rDNA gene have been used for parasite detection. On the other hand, the *g6pd* coding region, internal transcribed spacer 1 (ITS1) rRNA, and *hsp70* coding region have been more commonly used to identify the parasite at the species level [14, 32–38]. Conventional PCR and real-time PCR often followed by sequencing is increasingly being used for detection and identification of *Leishmania* spp. [6, 39]*.* Both methods are relatively laborious and time-consuming and they require PCR product manipulation, increasing the risk of laboratory contamination. Furthermore, the interpretation of the results requires also considerable skill and experience.

HRM is a relatively new real-time PCR coupled technique, with the first papers appearing in 2003 [40, 41]. The technique identifies nucleotide composition polymorphisms in real-time PCR products. The HRM methodology presents several attractive features: the whole process is performed in a closed-tube system (avoiding contamination in the laboratory) and it is relatively fast and cheap. The analysis may also be automated. The melting temperature generated depends on a range of factors, where GC-content, sequence, and length of the sequence are central. The melting curves can be objectively differentiated from each other by differences in melting temperature and shape [9].

For *Leishmania*, there are few studies utilizing HRM for detection and species identification. One of the earliest reports on the usefulness of HRM in differentiation of *Leishmania* species is by Nicolas et al. [42] who utilized polymorphisms in the coding sequence for minicircle kDNA to differentiate Eurasian and African species, *L.* (*L*.) *major*, *L.* (*L*.) *donovani* and *L.* (*L*.) *tropica*, and *L.* (*L*.) *infantum.* Later, Talmi-Frank et al. [43] utilized the ITS1 rRNA region to identify, distinguish and quantify Eurasian and African species, *L.* (*L*.) *infantum*/*L.* (*L*.) *donovani*, *L.* (*L*.) *aethiopica*, *L.* (*L*.) *tropica* and *L.* (*L*.) *major*. Both Nicolas et al. [42] and Talmi-Frank et al. [43] only targeted Eurasian and African species, making the approaches valuable in these endemic areas, although of limited value in other endemic areas and in a non-endemic settings where species from all endemic regions could be expected. Pita-Pereira et al. [33] also utilized the minicircle kDNA to discriminate among strains from the subgenus *L.* (*Viannia*) and *L.* (*L.*) *infantum* and *L.* (*L*.) *amazonensis*, making it an attractive methodology in an American setting to differentiate strains commonly causing cutaneous, mucocutaneous and visceral manifestations. Ceccarelli et al. [44] also demonstrated an ability to differentiate species of the subgenus *L.* (*Leishmania*) from *L.* (*Viannia*). HRM has also been used for detection of *Leishmania* in sand flies, where Aghaei et al. [45] used ITS1 to identify *L.* (*L*.) *tropica* in sand flies. Kuang et al. [46] utilized the *lack* gene to differentiate five Eurasian and African species from one American species, *L.* (*V*.) *braziliensis.* Although they showed its usefulness in clinical samples, the relatively few *Leishmania* species tested made it difficult to conclude if this target could be useful in other endemic areas. With increasing travel to several endemic regions, the correct diagnosis and species identification for the leishmaniases is of paramount interest. Hernandez et al. [36] utilized the ITS1 and *hsp70* to differentiate *L.* (*L*.) *mexicana*, *L.* (*L*.) *infantum*, *L.* (*L*.) *amazonensis*, *L.* (*V*.) *panamensis*, *L.* (*V*.) *guyanensis* and *L.* (*V*.) *braziliensis.* Although they found some ambiguities in species identification between the targets, they showed that ITS1 and *hsp70* had potential as diagnostic targets utilizing HRM. Zampieri et al. [14] showed, in a recent paper, that HRM targeting several polymorphic sites on the *hsp70* coding region could successfully be used to differentiate several Eurasian, African and American species. This makes it the most attractive target reported so far, especially useful in a non-endemic setting where the patient could have travelled to several endemic areas.

In this work, we describe a method for rapid detection and discrimination of most *Leishmania* species. We used the *aap3* coding sequence as target. *Leishmania aap3* coding sequences available in the GenBank and TriTryp databases were aligned to search for relatively conserved regions but present polymorphisms that enable the identification of different species. For this, the available coding sequences of some species from the subgenus *L.* (*Leishmania*) and *L.* (*Viannia*) were analyzed *in silico*. The coding sequence for *aap3* is present in two copies and organized *in tandem* in most of the *Leishmania* spp. genomes. For *L.* (*L*.) *donovani* and *L.* (*L*.) *amazonensis* a 98% identity has been described between the copies of coding regions and a 93% identity between these two species [16, 47]. In addition, *aap3* appeared conserved among other *Leishmania* species [17]. Although some species present only one *aap3* gene copy, for example *L.* (*V.*) *braziliensis*, this could be due the misannotation in the genome database. Considering these observations, the polymorphisms found between the two copies of the coding region did not affect the Tm analysis.

Amplicon 1 was able to discriminate the two strains causing visceral leishmaniasis from strains causing American cutaneous leishmaniasis. Furthermore, it was able to differentiate the American cutaneous strains and several of the strains of the subgenus *L.* (*Viannia*). The inability to differentiate the two visceral strains *L.* (*L*.) *donovani* from *L.* (*L*.) *infantum* was compensated with these strains having distinct Tm’s in amplicon 2 and amplicon 3. The same situation is true for the Eurasian cutaneous species: *L.* (*L*.) *major* and *L.* (*L*.) *tropica* were indistinguishable in amplicon 1, but had distinct Tm’s in amplicons 2 and 3. *Leishmania* (*L.*) *amazonensis* and *L.* (*L.*) *mexicana* are phylogenetically closely related species [48], and the difficulty in differentiating these species for diagnostic purposes has been described elsewhere [14]. Both amplicon 1 and 2 showed similar profiles for these two species, while amplicon 3 was able to distinguish them. Amplicon 2 was specifically designed for the subgenus *L.* (*Leishmania*), and there was no amplification of *L.* (*Viannia*) spp. High Cq values, observed for *L.* (*L.*) *amazonensis*, *L.* (*L*.) *mexicana* and *L.* (*L*.) *major* for amplicon 2, could be explained by primer-mismatches. Some caution should be taken when analyzing the result in a diagnostic setting, especially if negative. However, taken together the results from all amplicons can be considered to strengthen the diagnosis.

Diagnosing leishmaniasis relies on patient and travel history, clinical information (symptoms and clinical findings), and results from laboratory tests. Thus, for example, the inability of amplicon 1 to reliably distinguish *L.* (*L.*) *donovani* from *L.* (*L.*) *infantum*, in a patient with suspected visceral leishmaniasis is of little importance in a clinical setting, as the patient would receive the same treatment and follow-up regime regardless. But in an epidemiological setting, it is of importance to distinguish the species from each other to generate reliable data.

In general, multi-copy genes can be expected to yield a higher sensitivity in molecular diagnostics. The assays developed by Talmi-Frank et al. [43], Hernandez et al. [36] and Zampieri et al. [14], all report a limit of detection (LOD) of less than one parasite. Despite that *aap3* only comes in two copies we report a relatively good sensitivity with a LOD of 100 fg for amplicon 1 (just above 1 parasite) to 500 fg for amplicon 3 (approximately 5 parasites). This could probably be improved with a pre-amplification step.

**Fig. 3:**
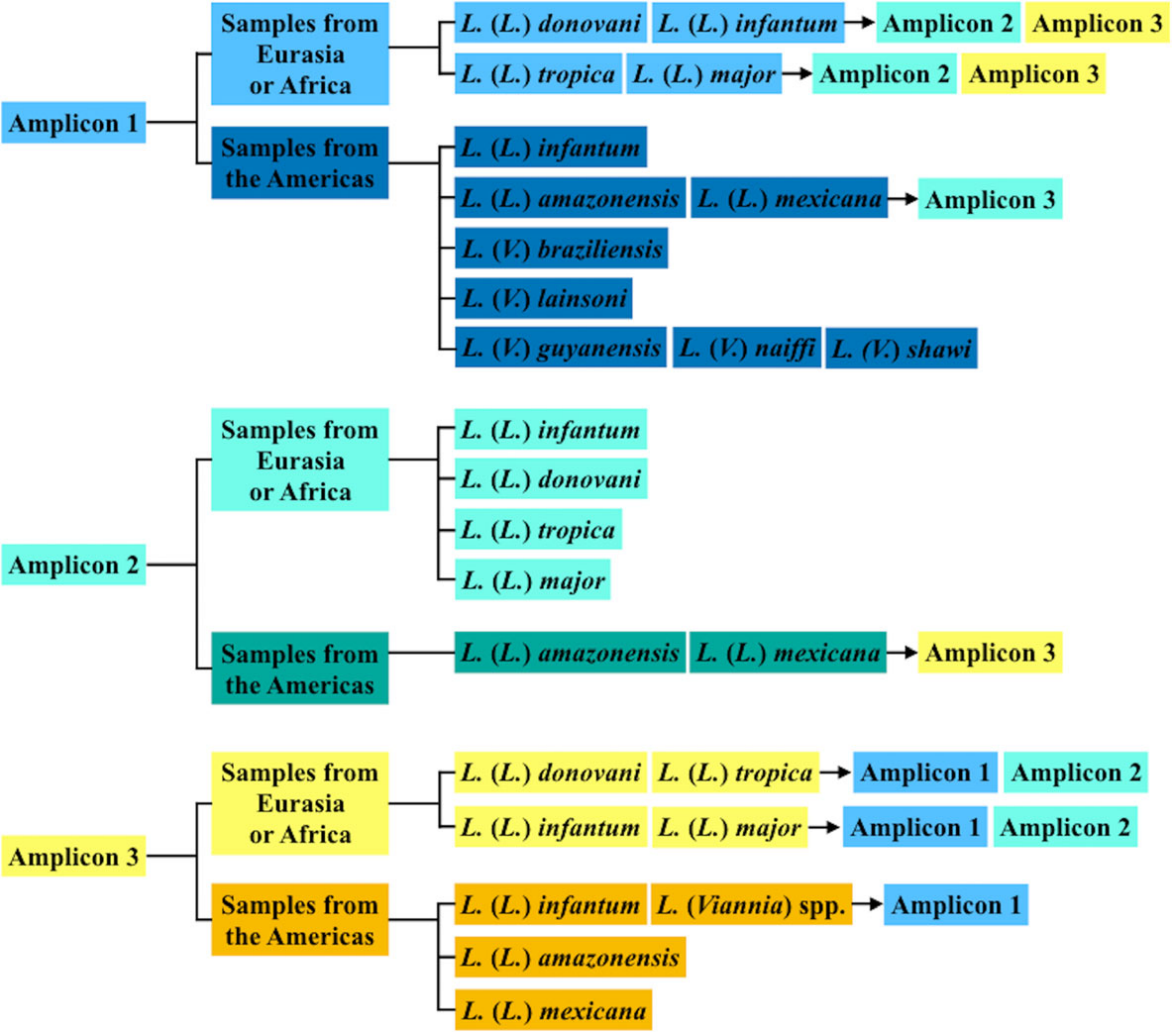
Proposed strategy for *Leishmania* species identification. Diagram of a proposed strategy using aap3-HRM for species identification. For VL patients from the Americas, amplicon 1 would suffice, while for VL patients from Eurasia and Africa we suggest the use of either amplicon 2 alone or amplicon 1 and 2. For patients with CL and MCL form the Americas, we suggest the use of both amplicon 1 and 3

Some HRM assays have found little evidence that DNA concentration of the initial template influences the Tm [36, 46]. However, the initial amount of DNA for this assay influenced the Tm for some of the species. This is in concordance with the findings of Zampieri et al. [14], who also found that initial DNA applied to the assay did affect the Tm for several strains. For the amplicons investigated in our study, this could lead to a misidentification for *L.* (*L.*) *mexicana* and *L.* (*L*.) *braziliensis* for amplicon 1. However, it should be noted that we propose the use of the three amplicons in the identification to strengthen the diagnostic validity and avoid species misidentification. A strategy for *Leishmania* species identification in patients with suspected leishmaniases is proposed in Fig. 3.

The validation of the target and the technique with clinical and experimentally infected samples from human, cat, mice and sand flies indicated a good correlation with other diagnostic targets and techniques. Not all amplicons were able to yield a result for all the samples, as could be expected due to differences in specificity and sensitivity of the primers, where amplicon 2 was not produced for any species belonging to the subgenus *L.* (*Viannia*). Despite the limited number of samples tested, the data indicate the potential of the *aap3*-HRM method to identify *Leishmania* species.

## Conclusions

Overall, the *aap3* coding sequence can be a promising target since it is specific and conserved for *Leishmania* spp. The design of the *aap3*-HRM protocol described is a relatively rapid, simple, sensitive and specific method to identify and distinguish several *Leishmania* spp*.* There is no need for sequencing or gel fractionation to analyze a PCR-product, minimizing the laboratory contamination as all the reactions are performed within a closed tube. The method can be automated, dispensing a trained technician to analyze the results. It also has the potential to quantify parasites present in samples, as it is a real-time PCR technique, able to detect a small number of parasites. In conclusion, the protocol described may offer a relatively low-cost and reliable method for detection and identification of *Leishmania* in biological and clinical samples.

## Additional files

Additional file 1: **Figure S1.** Agarose gel electrophoresis of PCR products. Reactions were performed using TopTaq Master Mix (Qiagen, Hilden, Germany) in a final volume of 25 μl with 200 nM of each primer and 25 ng of genomic DNA as a template. The PCR product was applied to a 3% agarose gel and stained with ethidium bromide. Conventional PCR products for standard strains and controls: A, amplicon 1 (expected 123 bp); B, amplicon 2 (expected 131 bp); C, amplicon 3 (expected 140 bp). (DOCX 8881 kb)

Additional file 2: **Figure S2.** Specificity using Cq values as parameter. Representative graph of Cq values obtained with HRM assays. The same amount of genomic DNA from all species was used as template to evaluate amplification efficiency. The samples used as negative controls are marked in red. Products generated in late Cq's (>30) were evaluated in the PikoReal software and revealed that Tm´s and melting profiles were different than for *Leishmania*. The fluorescence generated for these samples was due to unspecific amplification or noise. (DOCX 152 kb)

Additional file 3: Alignment of nucleotide sequences of *aap3* coding regions and primer localization. The underlined sequences indicate the position of the primers used and the grey boxes represent the variable regions found among the *Leishmania* strains based on *in silico* analysis. The numbers at the top of each amplicon are based on the position of the nucleotides in relation to the whole coding sequence in *L.* (*L*.) *amazonensis*. (DOCX 21 kb)

Additional file 4: **Figure S3.** Efficiency curves for all amplicons. Efficiency curves, slopes and R2 were calculated from four species for each amplicon using 25 ng, 5 ng and 5 pg of DNA from each *Leishmania* species. For amplicon 1 and 3 two species of *L.* (*Leishmania*) and two of subgenus *L.* (*Viannia*) were selected. Amplicon 2 only amplified *L.* (*Leishmania*), and strains from this subgenus were therefore selected. (DOCX 948 kb)

Additional file 5: Statistical analysis of melting temperatures (Tm’s) for all amplicons. (XLSX 16 kb)

## Abbreviations

aap3: Amino acid permease 3
BLAST: Basic local alignment search tool
CL: Cutaneous leishmanisis
Cq: Quantification cycle
DAT: Direct agglutination test
DNA: Deoxyribonucleic acid
g6pd: Glucose-6-phosphate dehydrogenase
HRM: High resolution melting analysis
hsp70: Heat-shock protein 70
ITS1: Internal transcribed spacer 1
kDNA: Kinetoplast DNA
LOD: Limit of detection
PCR: Polymerase chain reaction
SD: Standard deviation
SSU rDNA: Small subunit ribosomal DNA
VL: Visceral leishmaniasis
WHO: World Health Organization
