# Supplementary material for: Amino acid permease 3 (aap3) coding sequence as a target for Leishmania identification and diagnosis of leishmaniases using high resolution melting analysis
Source: Parasit Vectors. 2018 Jul 16;11:421. doi: 10.1186/s13071-018-2989-z (PMC6048756; doi:10.1186/s13071-018-2989-z)
Supplement: Supplementary file 1 — Figure S1. Agarose gel electrophoresis of PCR products. Reactions were performed using TopTaq Master Mix (Qiagen, Hilden, Germany) in a final volume of 25 μl with 200 nM of each primer and 25 ng of genomic DNA as a template. The PCR product was applied to a 3% agarose gel and stained with ethidium bromide. Conventional PCR products for standard strains and controls: A, amplicon 1 (expected 123 bp); B, amplicon 2 (expected 131 bp); C, amplicon 3 (expected 140 bp). (DOCX 8881 kb) [file 13071_2018_2989_MOESM1_ESM.docx]

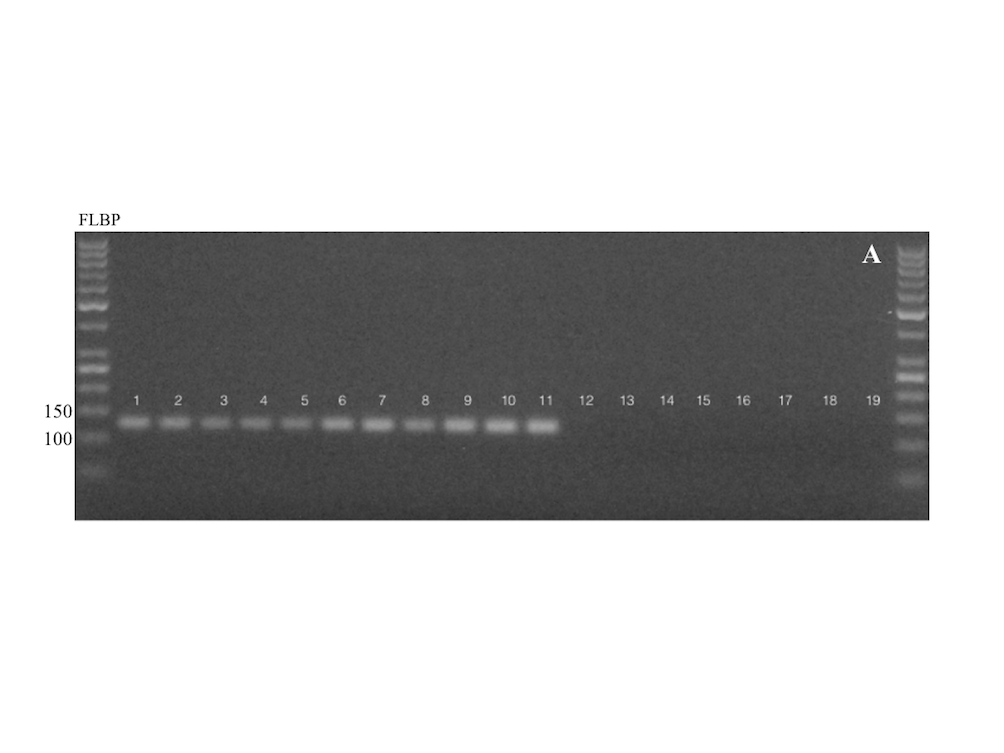


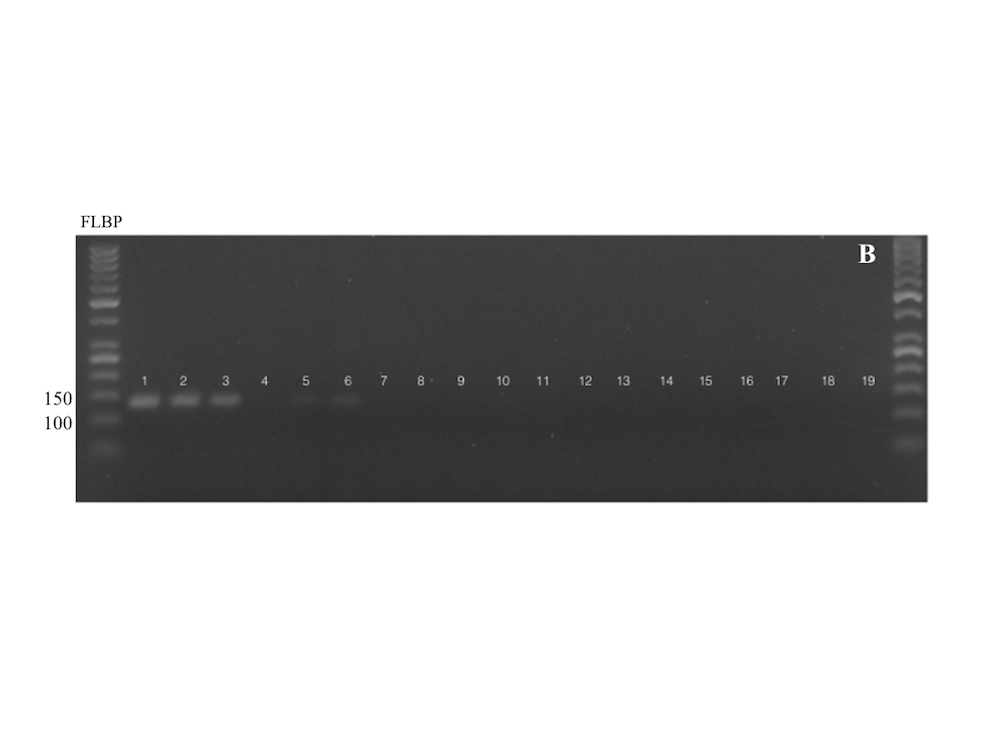


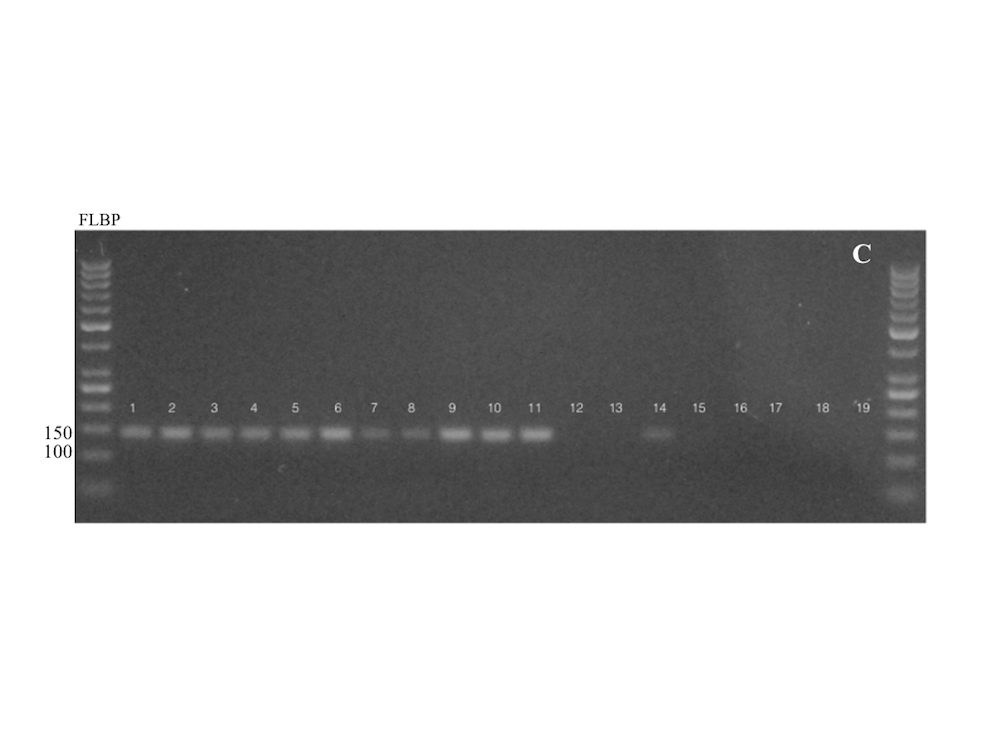


**Additional file 1: Figure S1**

Agarose gel electrophoresis of PCR products. Reactions were performed using TopTaq Master Mix (Qiagen, Hilden, Germany) in a final volume of 25 μL with 200 nM of each primer and 25 ng of genomic DNA as a template. The PCR product was applied to a 3% agarose gel and stained with ethidium bromide. Conventional PCR products for standard strains and controls: A – amplicon 1 (expected 123 bp); B - amplicon 2 (expected 131 bp); C – amplicon 3 (expected 140 bp).

| **Well** | **Description** |
| --- | --- |
| 1 | *L.* (*L.*) *donovani* |
| 2 | *L.* (*L.*) *infantum* |
| 3 | *L.* (*L.*) *tropica* |
| 4 | *L.* (*L.*) *major* |
| 5 | *L.* (*L.*) *amazonensis* |
| 6 | *L.* (*L.*) *mexicana* |
| 7 | *L.* (*V.*) *lainsoni* |
| 8 | *L.* (*V.*) *braziliensis* |
| 9 | *L.* (*V.*) *guyanensis* |
| 10 | *L.* (*V.*) *naiffi* |
| 11 | *L.* (*V.*) *shawi* |
| 12 | *T. brucei* |
| 13 | *T. cruzi* |
| 14 | *C. fasciculata* |
| 15 | *E. schaudinni* |
| 16 | Wistar rat |
| 17 | BALB/c mouse |
| 18 | Human |
| 19  FLBP | NTC  Fragment length in bp |
