# Supplementary material for: Amino acid permease 3 (aap3) coding sequence as a target for Leishmania identification and diagnosis of leishmaniases using high resolution melting analysis
Source: Parasit Vectors. 2018 Jul 16;11:421. doi: 10.1186/s13071-018-2989-z (PMC6048756; doi:10.1186/s13071-018-2989-z)
Supplement: Supplementary file 2 — Figure S2. Specificity using Cq values as parameter. Representative graph of Cq values obtained with HRM assays. The same amount of genomic DNA from all species was used as template to evaluate amplification efficiency. The samples used as negative controls are marked in red. Products generated in late Cq's (>30) were evaluated in the PikoReal software and revealed that Tm´s and melting profiles were different than for Leishmania. The fluorescence generated for these samples was due to unspecific amplification or noise. (DOCX 152 kb) [file 13071_2018_2989_MOESM2_ESM.docx]

1. **Cq-values for amplicon 1**





1. **Cq-values for amplicon 2**





1. **Cq-values for amplicon 3**





**Additional file 2: Figure S2:** Specificity using Cq values as parameter. Representative graph of Cq values obtained with HRM assays. The same amount of of genomic DNA from all species was used as template to evaluate amplification efficiency. The samples used as negative controls are marked in red. Products generated in late Cq´s (>30) were evaluated in the PikoReal software and revealed that Tm´s and melting profiles were different than for *Leishmania*. The fluorescence generated for these samples was due to unspecific amplification or noise.
