# Supplementary material for: Amino acid permease 3 (aap3) coding sequence as a target for Leishmania identification and diagnosis of leishmaniases using high resolution melting analysis
Source: Parasit Vectors. 2018 Jul 16;11:421. doi: 10.1186/s13071-018-2989-z (PMC6048756; doi:10.1186/s13071-018-2989-z)
Supplement: Supplementary file 3 — Alignment of nucleotide sequences of aap3 coding regions and primer localization. The underlined sequences indicate the position of the primers used and the grey boxes represent the variable regions found among the Leishmania strains based on in silico analysis. The numbers at the top of each amplicon are based on the position of the nucleotides in relation to the whole coding sequence in L. (L.) amazonensis. (DOCX 21 kb) [file 13071_2018_2989_MOESM3_ESM.docx]

**Amplicon 1 |...|....|....|....|....|....|....|....|....|....|....|....|....|....|....|....|....|....|....|....|....|....|....|....|..|**

**1676 1685 1695 1705 1715 1725 1735 1745 1755 1765 1775 1785 1798**

*L.* (*L.*) *amazonensis* **ATCCGCTACGTCTCCGCAATCGGTGTCTCCATGGTGCTGTATTTCGTTGCCACCATTGTGGTGCACTCGAGCATGCATGGGCTGAAGGAGGGTATGCGCGGTGACATGAAGTACTTCACCAGC**

*L.* (*L.*) *mexicana* **ATCCGCTACGTCTCCGCAATCGGTGTCTCCATGGTGCTGTATTTCGTTGTCACCATTGTGGTGCACTCGAGCATGCATGGGCTGAAGGAGGGCATGCGCGGTGACATGAAGTACTTCACCAGC**

*L.* (*L.*) *aethiopica* **ATCCGCTACGTCTCCGCAATCGGTGTCTTCATGGTGCTGTACTTCGCCGTCACTATTGTGGTGCACTCGAGCATGAATGGGATGAAGGAGGGCATGCGCGGTGACATGAAGTACTTCACCAGC**

*L.* (*L.*) *major* **ATCCGCTACGTCTCCGCAATCGGTGTCTTCATGGTGCTGTATTTCGCCGTCACTATTGTGGTGCACTCGAGCATGAATGGGATGAAGGAGGGCATGCGCGGTGACATGAAGTACTTCACCAGC**

*L.* (*L.*) *tropica* **ATCCGCTACGTCTCCGCAATCGGTGTCTTCATGGTGCTGTATTTCGCCGTCACTATTGTGGTGCACTCGAGCATGAATGGGATGAAGGAGGGCATGCGCGGTGACATGAAGTACTTCACCAGC**

*L.* (*L.*) *donovani* **ATCCGCTACGTCTCCGCAATCGGTGTCTTCATGGTGCTGTATTTTGCCGTCACTATTGTGGTGCACTCGAGCATGAATGGGCTGAAGGAGGGCATGCGCGGTGATATGAAGTACTTCACCAGC**

*L.* (*L.*) *infantum* **ATCCGCTACGTCTCCGCAATCGGTGTCTCAATGGTGCTGTATTTTGCCGTCACTATTGTGGTGCACTCGAGCATGAATGGGCTGAAGGAGGGCATGCGCGGGGACATGAAGTACTTCACCAGC**

*L.* (*V.*) *panamensis* **ATCCGCTACGTCTCCGCCATCGGCGTCACCATGGTGCTGTACTTTGTCGTCGTCATTGTGGTGCACTCCAGCACGAACGGCCTGAAGAAGGGCATGCGAGGCGACATGAAGTACTTCACCACG**

*L.* (*V.*) *braziliensis* **ATCCGCTACGTCTCCGCCATCGGCGTCATCATGGTGCTGTACTTTGTCGTCGTCATTGTGGTGCACTCCAGCACGAACGGCATGAGGGAAGGCATGCGAGGCGACATGAAGTACTTCACCACG**

**Amplicon 2 |...|....|....|....|....|....|....|....|....|....|....|....|....|....|....|....|....|....|....|....|....|....|....|....|....|....||**

**85 94 104 114 124 134 144 154 164 174 184 194 204 215**

*L.* (*L.*) *amazonensis* **GCCGTCACTAAATACCCGAGCGGTGAGCACGACAACCATCCCCTTAAAAGAGGAAGCCTGACAGACTCATCGAGCCACAATGGCAACGGTGCCGACGCCGCCAAGCCGGAGCGCAACATCATCTTCCGGTT**

*L.* (*L.*) *mexicana* **GCCGTCACTAAATACCCGAGCGGTGAGCACGACAGCCATCCCCTTAAAAGAGGAAACCTGACGGACTCATCGAGTCACAATGGCAACGGTGCCGACGCCGCCAAGCCGGAGCGCAACATCATCTTCCGGTT**

*L.* (*L.*) *aethiopica* **GCCGTCGGTAAACACCCGAGCGGCGTGCAGGGCAGCCACCCCCACAAAAACGGAAGCCTGACGGACTCATCAAGCCACAATGACAACGGTGCCGACGCCGCCAAGCCGAGCAGCAACATCATCTTCCGCTT**

*L.* (*L.*) *major* **GCCGTCGATAAACACCCGAGCGGAGAGCAGGGAAGCCACCTCCACAAAAGCGGAAGCCTGACGGACTCGTCAAGCCACAATGGCAACGGTGCCGACGCCGCCAAGCCGGAGCACAACATCATCCTCCGCTT**

*L.* (*L.*) *tropica* **GCCGTCGATAAACACCCGAGCGGCGTGCAGGGCAGCCACCCCCACAAAAACGGAAGCCTGACGGACTCGTCAAGCCACAATGACAACGGTGCCGACGCCGCCAAGCCGGGGCGCAACATCATCTTCCGCTT**

*L.* (*L.*) *donovani* **GCCGTCGATAAACACCCGAGCGGCGAGCAGGGCAACCATCTCCACAAAAACGGAAGCCTGACGGCCTCATCAAGCCACAATGAAAACGGCGCCGACGCCGCCAAGCCGGGGCGCAACATCATCTTCCGCTT**

*L.* (*L.*) *infantum* **GCCGTCGATAAACACCCGAGCGGCGAGCAGGGCAACCATCTCCACAAAAACGGAAGCCTGACGGCCTCATCAAGCCACAATGAAAACGGTGCCGACGCCGCCAAGCCGGGGCGCAACATCATCTTCCGCTT**

*L.* (*V.*) *panamensis* **ACGGGCGGTGTTGACCTGAGCGACGGGCAGGTGAAGCGCCCCCTCCACAGTGGGAGCCCGACGGAGTCCACTGGTCACCACAACGACAG---CGACGTCCAGAAGCGGCAGCCCAACATCATCTTCCGCTT**

*L.* (*V.*) *braziliensis* **ACGGGCGGTGTTGACCCGAGCGACGGGCAGGTGAAGCGCCCTCTCCACAGTGGGAGCCCGACGGAGTCCACTGGTCACCACAACGACAG---CGACGTCCAGAAGCGGCAGCCCAACATCATCTTCCGCTT**

**Amplicon 3 |...|....|....|....|....|....|....|....|....|....|....|....|....|....|....|....|....|....|....|....|....|....|....|....|....|....|....|....|**

**510 519 529 539 549 559 569 579 589 599 609 619 629 639 649**

*L.* (*L.*) *amazonensis* **GGCGGTCGCCTACATCAGCGCCGTCAGCAGCCTCATCACGCCGATCCTCGAGAAGTCGCCCGGCACGCCCGCATACCTGCTCACCACCTCCGGCAACCGCCTGATCACGAGCCTGATATGGCTCGTGTTCATGGTGCCCG**

*L.* (*L.*) *mexicana* **GGCGGTCGCCTACATCAGCGCCGTCAGCAGCCTCATCACGCCGATCCTCGAGAAGTCGCCCGGCACGCCCGCGTACCTGCTCACCACCTCCGGCAACCGCCTGATCACGAGCTTGGTATGGCTCGTGTTCATGGTGCCCG**

*L.* (*L.*) *aethiopica* **GGCGGTCGCCTACATCAGCGCCGTCAGCAGCCTCATCACACCGATCCTCGAGAAGTCGCCCGGCACGCCCGCGTACCTGCTAACCACCTCCGGCAACCGCCTGATCACGAGCCTGATATGGCTCGTGTTCATGGTGCCAG**

*L.* (*L.*) *major* **GGCGGTCGCCTACATCAGCGCGGTCAGCAGCCTCATCACACCGATCCTTGAAAAGTCGCCCGGCACGCCCGCGTACCTGCTAACCACCTCCGGCAACCGCGTGATCACGAGCCTGATATGGCTCGTGTTCATGGTGCCAG**

*L.* (*L.*) *tropica* **GGCGGTCGCCTACATCAGCGCCGTCAGCAGCCTCATCACACCGATCCTCGAGAAGTCGCCCGGCACACCCGCGTACCTGCTAACCACCTCCGGCAACCGCCTGATCACGAGCCTGATATGGCTCGTGTTCATGGTGCCGA**

*L.* (*L.*) *donovani* **GGCGGTCGCCTACATCAGCGCCGTCAGCAGCCTCATCACACCGATCCTCGAGAAGTCGCCCGGCACGCCCGCGTACCTGCTAACCACCTCCGGCAACCGCCTGATCACGAGTCTGATATGGCTCGTGTTCATGGTGCCAG**

*L.* (*L.*) *infantum* **GGCGGTCGCCTACATCAGCGCCGTCAGCAGCCTCATCACACCGATCCTCGAGAAGTCGCCCGGCACGCCCGCGTACCTTCTAACCACCTCCGGCAACCGCCTGATCACGAGCCTGATATGGCTCGTGTTCATGGTGCCAG**

*L.* (*V.*) *panamensis* **TGCCGTGGCCTACATCAGCGCCGTCAGCAGCCTGATCTCGCCGATCCTCGAGAAGTCCCCTGGGACGCCGGCGTTTCTCCTGACCACTGCCGGCAACCGCTGCATCACCAGCCTTATCTGGCTCGTGCTCATGGTGCCAG**

*L.* (*V.*) *braziliensis* **TGCCGTGGCCTACATCAGCGCCGTCAGCAGCCTGATCTCGCCGATCCTCGAGAAGTCCCCTGGGACGCCGGCGTTTCTCCTGACCACTGCCGGCAACCGCTGCATCACCAGCCTTATCTGGCTCGTGCTCATGGTGCCAG**

**Additional file 3: Alignment of nucleotide sequences of *aap3* coding regions and primer localization.**

The underlined sequences indicate the position of the primers used and the grey boxes represent the variable regions found among the *Leishmania* strains based on *in silico* analysis. The numbers at the top of each amplicon are based on the position of the nucleotides in relation to the whole coding sequence in *L. (L.) amazonensis*.
