# Supplementary material for: Amino acid permease 3 (aap3) coding sequence as a target for Leishmania identification and diagnosis of leishmaniases using high resolution melting analysis
Source: Parasit Vectors. 2018 Jul 16;11:421. doi: 10.1186/s13071-018-2989-z (PMC6048756; doi:10.1186/s13071-018-2989-z)
Supplement: Supplementary file 4 — Figure S3. Efficiency curves for all amplicons. Efficiency curves, slopes and R2 were calculated from four species for each amplicon using 25 ng, 5 ng and 5 pg of DNA from each Leishmania species. For amplicon 1 and 3 two species of L. (Leishmania) and two of subgenus L. (Viannia) were selected. Amplicon 2 only amplified L. (Leishmania), and strains from this subgenus were therefore selected. (DOCX 948 kb) [file 13071_2018_2989_MOESM4_ESM.docx]

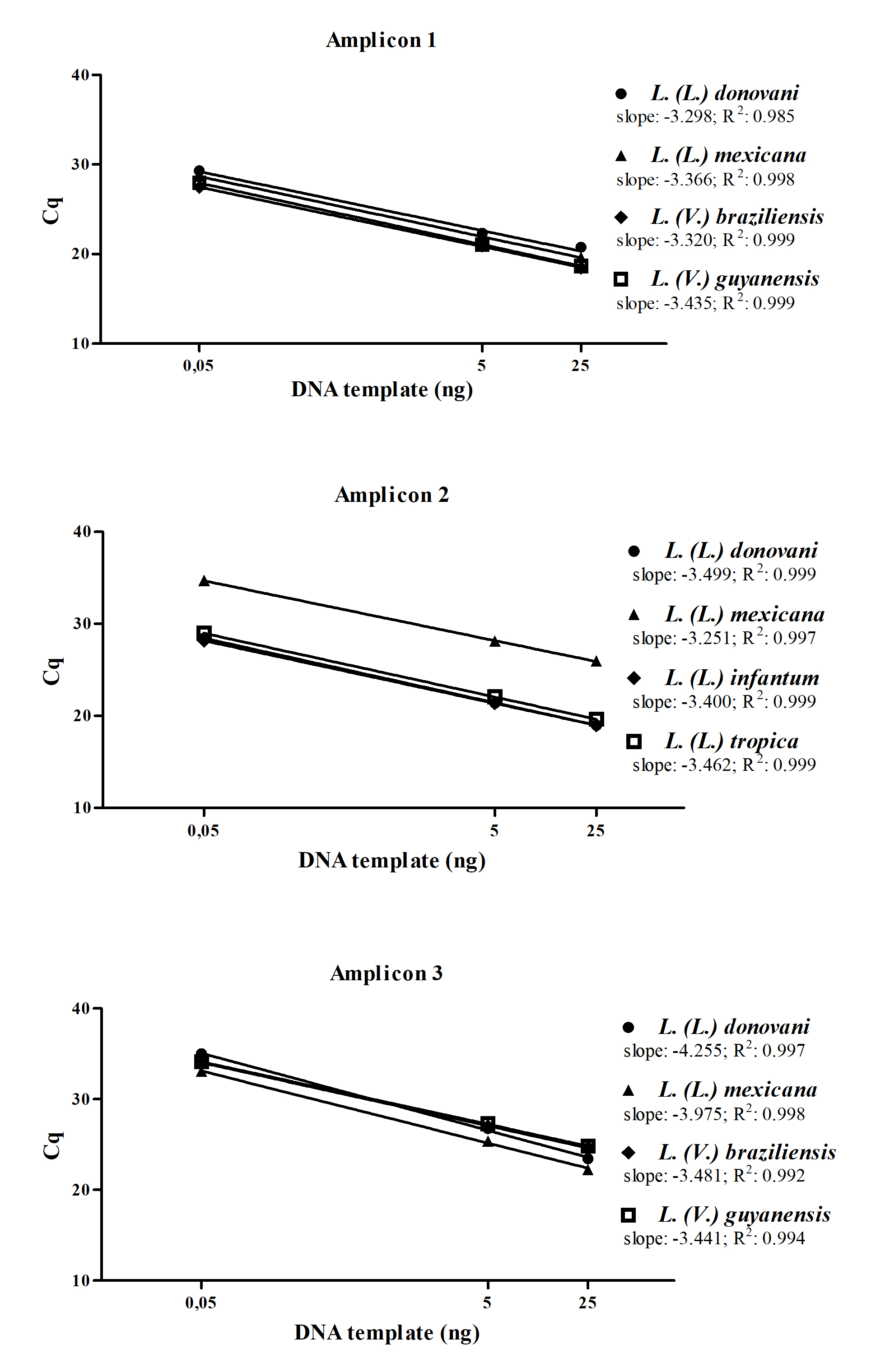


**Additional file 4: Figure S3: Efficiency curves for all amplicons.**

Efficiency curves, slopes and R^2^ were calculated from four species for each amplicon using 25 ng, 5 ng and 5 pg of DNA from each *Leishmania* species. For amplicon 1 and 3 two species of *L.* (*Leishmania*) and two of *L.* (*Viannia*) subgenus were selected. Amplicon 2 only amplified *L.* (*Leishmania*), and strains from this subgenus were therefore selected.
